# Breakthroughs in microbiology made possible with organoids

**DOI:** 10.1371/journal.ppat.1010080

**Published:** 2021-11-24

**Authors:** Carlos J. Ramírez-Flores, Laura J. Knoll

**Affiliations:** University of Wisconsin-Madison, Madison, Wisconsin, United States of America; Mount Sinai School of Medicine, UNITED STATES

To study the host–pathogen interactions with medical and veterinary relevance, researchers have recently applied organoid technology. Organoids revolutionized biomedical research in the last decade due to their ability to recapitulate the physiological properties of whole organs in cell culture. For the field of microbiology, organoids have allowed the study of previously recalcitrant pathogens that would only replicate in their natural host. Nowadays, a large list of viruses, bacteria, fungi, and parasites have been studied using organoids. Here, we describe breakthrough studies that have had or are predicted to have major impacts in the microbiology field, which would not have been possible without the use of organoid technology.

## Organoids: What are they, and what are they used for?

**Fig 1:**
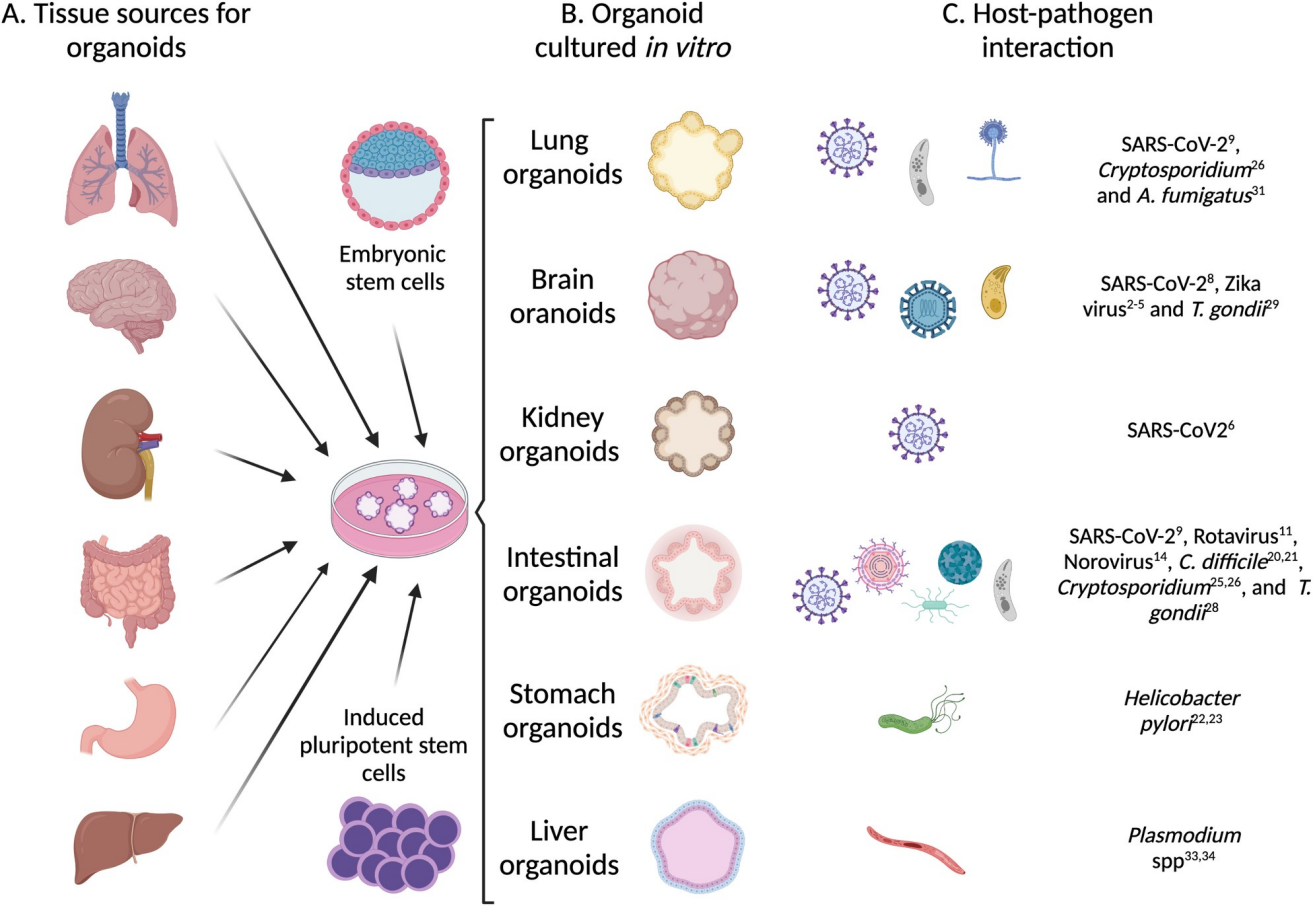
Applications of organoids in the study of host–pathogen interactions. **(A)** Organoids can be derived from a wide range of humans or animal tissues as well as embryonic and induced pluripotent stem cells. **(B)** Biopsies of the tissues are cultured in vitro for the formation of organoids. Organoids established in culture can resemble the architecture and biological function of its tissue. **(C)** Organoids can be infected by diverse pathogens, mimicking the in vivo host interaction, and used subsequently in applied research. This figure was created using Biorender.com, agreement number BE234AXCJN. SARS-CoV-2, Severe Acute Respiratory Syndrome Coronavirus 2.

While the two-dimensional (2D) culture of host cells has provided valuable insights for the microbiology field, they lack many of the components needed to recreate the natural host environment for investigating pathogens, such as the microbiota, cellular complexity, and polarity. Organoids are cultured from stem cells and form three-dimensional (3D) structures that mimic the organ, including multiple cell types, protein expression, and functions such as absorption, barrier function, and nutrient uptake (Fig 1). Human intestinal, lung, kidney, brain, and liver organoids have all been used to study host–microbe interactions and to test drug potential treatments. Because the brain has a particularly complex structure and strict restrictions exist for obtaining human samples, human brain organoids (HBOs) have been a significant advance for the study of microbes that infect the brain.

## What rapid breakthroughs occurred studying pandemic viruses?

Zika virus (ZIKV) spreads by the bite of an infected *Aedes* spp. mosquito causing mild flu symptoms in adults and congenital Zika syndrome in newborns. Congenital Zika syndrome includes several birth defects but is most known for small infant head size called microcephaly. Zika infection of HBO allowed the mechanism by which ZIKV affects the neurons to be determined [1,2] as well as the evaluation of potential treatments [3]. ZIKV-infected HBOs have shown that replication of the virus killed neural precursors that led to microcephaly [1,2]. The microcephaly model of ZIKV in HBO further showed thinning of the cortices and impaired cortical expansion, which also lead to HBO size reduction [4]. Neurosphere and HBO infection with Brazilian and African ZIKV strains showed larger reductions in the proliferative zones, greater disruption of the cortical layers, and an increased number of apoptotic cells with the Brazilian compared to the African ZIKV strain [5]. Other advantages and limitations of the HBO to study ZIKV over other in vitro models as well as advances to understand the affections in HBO that resemble microcephaly were recently summarized [6].

The study of Severe Acute Respiratory Syndrome Coronavirus 2 (SARS-CoV-2) marked a breakthrough in the viral pathogenesis field with the rapid modeling of the infection using organoids. Kidney, small intestine, lung, and cerebral organoids have helped to study the multi-organ infection/damage observed by SARS-CoV-2 [7–10]. SARS-CoV-2 causes Coronavirus Disease 2019 (COVID-19). COVID-19 shows influenza-like symptoms and injury in the airways, gastrointestinal tract, and the central nervous system (CNS), causing meningitis/encephalitis. Modeling of these tissues in organoids allowed researchers to determine the differential expression of cytokines and chemokines in colonic organoids, neuronal death, hypermetabolic state and hypoxia in HBO, and chemokine production and responsiveness to drug treatment in lung organoids [9,10]. SARS-CoV-2 infection of human kidney organoids and HBO confirmed viral binding to the angiotensin converting enzyme 2 receptor in 3D culture [7,10]. These examples show how organoid technology allowed researchers to efficiently recapitulate host–pathogen interactions to study the physiopathology of viruses and how we can cope with future pandemics.

## What are the breakthroughs in gastrointestinal viral infections?

The study of human enteric viruses, such as rotavirus, norovirus, adenovirus, and astrovirus, have all been advanced using intestinal organoid technology [11]. Rotavirus and norovirus are responsible for food and waterborne diarrhea, causing together >1 billion cases and >500,000 deaths per year in children <6 years old (World Human Organization). A breakthrough for gastrointestinal viruses was obtained by the reproducible cultivation of stool-isolated rotavirus in human intestinal organoids (HIOs) [12]. This study showed rotavirus replication in mesenchymal as well as the epithelial cells. In human biliary liver organoids, robust rotavirus replication was blocked by antiviral drugs and neutralizing antibodies [13]. For previously uncultivable norovirus, HIO supported replication and modeling multiple human variants [14]. During norovirus infection of HIO, researchers discovered essential cofactors (e.g., bile acids, histo-blood group antigens, and divalent cations), overexpression of genes, and response to type I and III interferons [14–16].

## What breakthroughs have occurred studying gastrointestinal bacteria?

Enteric bacterial infections remain a health challenge. The study of pathology caused by bacteria in immortalized 2D cultures, ex vivo tissues, and animal models has contributed to the knowledge in this field. However, 2D cell lines lack the structural complexity of tissues, ex vivo tissues have a limited life span, and animal models have many pathophysiological and immune response differences from humans. Because of these limitations, HIOs that resemble the intestinal environment are emerging as models for bacterial infections.

Multiple enteric bacteria, such as *Escherichia coli*, *Salmonella*, and *Listeria*, have been extensively studied using organoids to provide valuable insights and expand our knowledge of bacteria–host interaction [17,18]. Foundational experiments creating an anaerobic environment for examining interactions of anaerobic bacteria with host epithelium have been performed in HIOs. *Clostridium difficile*, the agent responsible for 25% of the nosocomial diarrhea cases, is an obligate anaerobe and notoriously difficult to study. Microinjection of *C*. *difficile* into the lumen of the HIO resulted in infection, toxin production, and consequent paracellular barrier disfunction and altered mucus oligosaccharide composition [19,20]. As another example, *Helicobacter pylori* colonize the gastric mucosa and is the main cause of peptic ulcers, chronic gastritis, and gastric cancer. Microinjection of *H*. *pylori* into gastric organoids can emulate changes and pathological events during its infection, such as inflammation and regulation of tight junctions [21,22].

## What are the current breakthroughs using organoids in the parasites field?

*Cryptosporidium* is an intracellular parasite that causes diarrhea and gastroenteritis in vertebrates including humans. In the past, short-term (up to 5 days) infection and incomplete propagation was supported in a 2D culture of human intestinal epithelial cells [23]. Recently, 3D systems have been used to successfully sustain the infection from oocysts and generate damage in colon explants, providing evidence that the parasite could induce cancer [24]. *Cryptosporidium parvum* can infect human small intestinal and lung organoids where it can successfully develop for up to 28 days [25]. Addition of an air–liquid interface to the culture system allowed for life cycle completion, including the production of oocysts that were infectious to mice [26].

Some parasites have strict species specificity to their life cycles, which has hindered their study in vitro. Organoids have opened an avenue for those parasites with host-specific requirements. *Toxoplasma gondii* can infect any warm-blooded vertebrate, but its sexual cycle is restricted to the feline intestine. The specificity of *T*. *gondii* sexual development in cat intestinal organoid-derived monolayers was determined. Cat intestinal cells supplemented with linoleic acid supported early sexual development of *T*. *gondii* in vitro [27]. To model *T*. *gondii* human brain infection, HBOs were infected with the rapidly replicating form called a tachyzoite, which then spontaneously transformed into the chronic cyst form called a bradyzoite [28]. Changes in transcriptomics related to parasite invasion and replication were also detected in these HBOs. Recently, a multispecies organoid platform was successfully modeled to coinfect *T*. *gondii* with *Giardia duodenalis* by using organoids or organoid-derived monolayers of various host species [29].

## What discoveries have been made culturing fungi in organoids?

The study of fungi in traditional 2D cultures can miss key signaling events that occur during host–fungus interactions. To capture the complexity of the lung, researchers used human lung organoids to develop a model of the bronchiole including an airway, vascular, and extracellular matrix components [30]. This model contained a clickable extension to facilitate volatile compound communication between the microbes and host. Immune cell recruitment and leukocyte extravasation were examined in this model after coinfection with the fungal pathogen *Aspergillus fumigatus* and the bacteria *Pseudomonas aeruginosa*. Greater inflammatory responses were seen in these bronchioles when they were in contact with volatiles from both pathogens, compared to either monoculture, showing volatile communication between the kingdoms [30].

## What is next?

Organoid cultures of *Plasmodium* spp., which are well known as the causative agents of malaria, are examples of some progress but continued limitations that must be addressed to model the infection successfully. These limitations include long-term culture, poor infectability, size of the organoids for consistent infection, cell function, presence of immune cells, difficulty to test drugs, and parasites being trapped within the matrix [31]. To date, the liver stage has been studied using rodent-infecting parasite *Plasmodium berghei* in liver spheroids, resulting in infective liver stages called merozoites [32]. Liver spheroids derived from simian and human hepatocytes supported the complete liver stage of *Plasmodium cynomalgi* and *Plasmodium vivax*, starting with the sporozoite stage and finishing with the release of merozoites capable of invading erythrocytes in vitro [33]. Those results have shown that the use of organoids in the malaria field is promising for the study of *Plasmodium* species that infect humans.

Organoids are still in their infancy and under constant development. Improvements to the system are required to keep revolutionizing the field. Integration of immune cells, low oxygen conditions, and microbiota to emulate the host microenvironment are progressing as well as adding variability of the structure and size. Organoid–pathogen interaction of other microbes should be investigated further to fill the gaps from 2D systems. Modeling infection will be useful to know mechanisms and factors of host/pathogen specificity during the life cycle of microbes.
